# Multi‐omics insights into Alzheimer's disease stratification using a microglial polygenic risk score

**DOI:** 10.1002/alz70855_102432

**Published:** 2025-12-23

**Authors:** Masataka Kikuchi, Akinori Miyashita, Yu Hirota, Norikazu Hara, Mai Hasegawa, Yasufumi Sakakibara, Michiko Sekiya, Koichi M. Iijima, Takeshi Ikeuchi

**Affiliations:** ^1^ Brain Research Institute, Niigata University, Niigata, Niigata, Japan; ^2^ National Center for Geriatrics and Gerontology, Obu, Japan; ^3^ Brain Research Institute, Niigata University, Niigata, Japan

## Abstract

**Background:**

Recent single‐cell omics studies have highlighted the diverse gene expression profiles of microglia in response to stress environments. Microglia are known to transition into a reactive state with phagocytic activity in response to Aβ accumulation in the brain. However, individuals with TREM2 gene mutations exhibit reduced induction of reactive microglia. This microglia‐centered pathological mechanism is a recognized feature of Alzheimer's disease (AD). Nevertheless, single‐gene mutations such as TREM2 rarely account for the complexity of AD pathology. Instead, polygenic influences involving multiple genetic variations have been proposed. To quantify these polygenic effects, we developed a microglial polygenic risk score (PRS) and used it to stratify AD patients. We then performed multi‐omics analyses of postmortem brain samples to investigate pathological differences between PRS‐defined groups.

**Method:**

Postmortem brain samples from 100 individuals with Braak stage information for senile plaques (SP) and neurofibrillary tangles (NFT) were analyzed. Whole‐genome sequencing and bulk RNA sequencing (RNA‐seq) of the frontal cortex were conducted. Additionally, a subset of 15 samples (8 controls, 7 AD patients) underwent single‐nucleus RNA‐seq (snRNA‐seq) and spatial transcriptomics to achieve higher‐resolution analyses.

**Result:**

AD patients were stratified into low‐PRS and high‐PRS groups based on microglial PRS, followed by bulk RNA‐seq analysis. Differential expression analysis identified 112 genes associated with autophagy and inflammation. To validate these findings at the single‐cell level, snRNA‐seq revealed distinct microglial clusters in both PRS groups. Comparative analyses of gene expression profiles in 3 low‐PRS and 4 high‐PRS AD patients showed significant variability in microglial reactivity to Aβ. These findings, supported by spatial transcriptomics, suggest heterogeneity in microglial responses within the same AD pathology depending on genetic predisposition.

**Conclusion:**

PRS‐based stratification of AD patients reveals that microglial reactivity varies according to the strength of genetic predisposition, even among individuals with the same pathological diagnosis. Stratification by genetic factors, such as PRS, may pave the way for novel therapeutic strategies tailored to distinct genetic profiles.

